# Research involving subjects with Alzheimer’s disease in Italy: the possible role of family members

**DOI:** 10.1186/s12910-015-0009-9

**Published:** 2015-03-04

**Authors:** Corinna Porteri, Carlo Petrini

**Affiliations:** Bioethics Unit, IRCCS Centro San Giovanni di Dio Fatebenefratelli, Via Pilastroni 4, 25125 Brescia, Italy; Bioethics Unit, Istituto Superiore di Sanità, Viale Regina Elena 299, 00161 Rome, Italy

**Keywords:** Alzheimer’s disease, Clinical trials, Competence, Legal representative, Family members

## Abstract

**Background:**

Alzheimer’s disease is a very common, progressive and still incurable disease. Future possibilities for its cure lie in the promotion of research that will increase our knowledge of the disorder’s causes and lead to the discovery of effective remedies. Such research will necessarily involve individuals suffering from Alzheimer’s disease. This raises the controversial issue of whether patients with Alzheimer’s disease are competent to give their consent for research participation.

**Discussion:**

We discuss the case of subjects with Alzheimer’s disease who may have impaired decision-making capacity and who could be involved in research protocols, taking into consideration aspects of the Italian normative framework, which requires a court-appointed legal representative for patients who are not able to give consent and does not recognise the legal value of advance directives. We show that this normative framework risks preventing individuals with Alzheimer’s disease from taking part in research and that a new policy that favours research while promoting respect for patients’ well-being and rights needs to be implemented.

**Summary:**

We believe that concerns about the difficulty of obtaining fully valid consent of patients with Alzheimer’s disease should not prevent them from participating in clinical trials and benefiting from scientific progress. Therefore, we argue that the requirement for patients to have a legal representative may not be the best solution in all countries and clinical situations, and suggest promoting the role of patients’ family members in the decision-making process. In addition, we outline the possible role of advance directives and ethics committees.

## Background

Alzheimer’s disease (AD) is a very common, progressive and still incurable disease. It is estimated that AD along with other dementias with similar clinical profiles affect an estimated 35.6 million people—around 0.5% of the global population [1]. Although some studies show a substantial decrease in dementia prevalence [2] and a substantial increase in cognitive performance and activities of daily living in elderly people [3] in some high-income countries, ‘even with a small decrease in incidence and prevalence, population ageing will still double the numbers with dementia worldwide in the next generation’ [4]. These estimates are confirmed by the World Health Organization [5].

In Italy, there are approximately 700,000 people with AD and 80,000 new cases per year [6]. Though the capacity to diagnose this disease even at a prodromal stage has continued to improve [7], effective opportunities for treatment and prevention are still lacking. Current drugs (i.e. cholinesterase inhibitors and memantine) can provide symptomatic relief for a number of patients but cannot delay disease progression. Unfortunately, these drugs, together with cognitive training and rehabilitation, for which there is still no indication of any significant benefit [8], are the only real options available today.

This disease has devastating effects on the lives of patients and their caregivers and has important social and economic implications. The future possibilities of a cure lie in research that can increase our knowledge of the aetiology of the disease and promote the discovery of effective remedies, both in terms of biological changes and clinical improvements. Such research will necessarily involve individuals suffering from AD. This raises the controversial issue of whether patients with AD are competent to give consent for research participation.

In this paper, we discuss the situation of individuals with AD who may have impaired decision-making capacity and who might be involved in research protocols, taking into consideration aspects of the Italian normative framework, which risks preventing this population from participating in research. We suggest a possible way to aid research while promoting the well-being and rights of patients involved in clinical trials.

We chose to consider AD because it is the most common form of dementia, accounting for 50 to 80% of all dementia cases [1], and is currently the object of the largest research investments and the most extensive literature. Nevertheless, our discourse can be applied to other dementias too.

## Discussion

### Consent for research by incompetent patients

Informed consent is a fundamental ethical and legal requirement of biomedical research to ensure that participants are not objects of exploitation but, on the contrary, are considered autonomous subjects entitled to be informed and make decisions for themselves. In cases where the interested person is judged unable to give consent, international guidelines (WMA Helsinki Declaration [9]; CIOMS International Ethical Guidelines for Biomedical Research Involving Human Subjects [10]) and European regulations (Directive 2001/20/EC [11]; Additional protocol to the convention on human rights and biomedicine, concerning biomedical research [12]; Regulation (EU) No 536/2014 [13]) require consent from the person’s legal representative. Directive 2001/20/EC has been transposed into national laws by member states and, since its enforcement, has been the common framework for clinical trials in the European Union. In Italy, the directive has been enforced by the Legislative Decree no. 211, 24 June 2003 [14].

The new Regulation (EU) no 536/2014, which came into force on 16th June 2014 (but will not be applicable until 6 months after a new EU portal and database have become fully functional, and not before 28 May 2016), repeals that directive and establishes a new set of harmonised rules that member states should apply in clinical trials. Both the directive and the regulation allow the inclusion of incapacitated adults (who have not given or not refused informed consent before the onset of their incapacity) only if ‘the informed consent of their legally designated representative has been obtained’ (Regulation EU 536/2014, art. 31.a).

Based on the European normative framework, national legislation specifies which persons can act as a patient’s representative: ‘Regarding the rules concerning the determination of the legally designated representatives of incapacitated persons and minors, those rules diverge in member states. It should therefore be left to member states to determine the legally designated representatives of incapacitated persons and minors’ (Regulation EU 536/2014, Whereas 27).

This implies that approaches to the appointment of a patient’s legal representative may differ across member states depending on the legal, cultural and political background of each European country, giving rise to procedures of differing complexity that may affect biomedical research. Information on European legislations has been compiled [15-17] and the issue has been discussed in a significant number of commentaries [18-22].

The main distinction can be drawn between countries where the legal representative must be appointed by the court, as in Austria, Germany and Italy, and countries that, although with some differences in the implementation of the provision, have adopted a more pragmatic procedure involving a cascade of measures to arrive at consent on behalf of the subject, ranging from the authorised legal representative to family members and close persons. These countries include member states that specify a hierarchy of family members to be contacted, such as Belgium, Hungary, The Netherlands and Norway, as well as countries with more general provisions, such as Denmark, Finland, France, Ireland, the United Kingdom, Spain and Sweden, where there must be a close relationship between the subject and their representative. In France, authorisation is required from a judge if an ethics committee rules that the research imposes serious risks to the participants. In countries like the United Kingdom, Denmark and Ireland, professional representatives may give consent on behalf of the patient provided they are not connected to the clinical trials.

In Italy, a legal representative has to be appointed by the court in a case-by-case manner to comply with the rules of the Italian Civil Code [23]. This procedure applies both for biomedical research and for health-related treatments. Before the approval of Law 6/2004 [24] that instituted guardianship [*amministratore di sostegno*] and modified Title XII of the Italian Civil Code, the only possible legal representative for an incompetent adult was the *tutore*. A *tutore* could be appointed through a slow, costly and permanent procedure either for acts of extraordinary administration (i.e. disqualification) or for acts of both extraordinary and ordinary administration (i.e. proscription). Both measures have a serious impact on the person concerned, whose interests are consequently handled by the appointed representative in accordance with whichever measure is adopted, and both have largely fallen into disuse. Law 6/2004 permits a quicker and less costly way to appoint a legal representative for vulnerable individuals and facilitates an important change in the way that vulnerable people are viewed [25]. In fact, the traditional institutions of disqualification and proscription completely deprived a person of her legal rights, while guardianship focuses on the person’s care and assistance needs. Moreover, the person in question may indicate in advance her representative, and she maintains the capacity to act in all aspects that are not explicitly included in the responsibilities of the *amministratore di sostegno*.

For the above reasons, in health-related issues, the *amministratore di sostegno* is a better option than the *tutore* for circumstances in which a person clearly needs a legal representative.

Nevertheless, there are some important considerations with respect to AD patients’ participation in biomedical research, namely: i) the case of appointing a legal representative just for research participation; ii) the case of uncertain capacity; and iii) the case of collaborative research.

### The case of appointing a legal representative just for research participation

Most people with AD who attend memory clinics and are potential research subjects do not have a legal representative at the time they are asked to take part in research. Previous experiences show that the requirement of a legal representative as a condition for the enrolment of patients in a clinical trial is a major obstacle to research and may prevent the implementation of the trial. This was the case in the AdCare study, a non-profit, randomised, placebo-controlled, double-blind, multicentre trial coordinated by the Italian National Institute of Health, which aimed to evaluate the long-term safety and efficacy profiles of atypical and conventional antipsychotic drugs. The study planned to include 19 clinical centres with the enrolment of 1000 outpatients suffering from AD. According to Italian law, in cases where the research team evaluated a patient as unable to give informed consent, a legal representative designated by the court was asked to provide consent on behalf of the patient. This requirement led to serious difficulties in the enrolment of subjects; consequently, the study was prematurely suspended. From February 2009 to April 2010, only 83 patients gave informed consent to participate in the trial. Fifty-six patients (68%) were included with consent given by a legal representative, while 27 patients (32%) were considered able to provide their own informed consent after an assessment of their competence [26]. A sub-study on legal agency was performed at one of the centres involved in the AdCare study and involved 172 patients. The results showed that only three participants were acquainted with Law 6/2004. More than half of the patient/relative couples chose not to initiate the procedure for the appointment of a legal representative, while 46% applied for the appointment. The mean time interval between the presentation of the law by the research team and the application to the court was 2 months. The mean time interval between the application to the court and the completion of the appointment was 4 months (double the maximum time prescribed by the law). Seventy percent of applications resulted in the appointment of a legal representative. The study found that the subjects who decided to apply for a legal representative were usually younger, had been suffering from dementia for longer and had fewer than two children [18].

The above evidence shows that the request for a legal representative for patients’ participation in clinical trials is not really practicable within the current Italian legal framework for at least two reasons: first, the time needed to appoint the legal representative, which inevitably slows down the research schedule, and second, the attitudes and sensitivity of the patients’ family members. The latter reason should be seriously considered. As for the AdCare study, clinical practice shows that very few patients with dementia have a legal representative. Moreover, it is uncommon to initiate the legal procedure for the appointment of a representative unless there are compelling reasons to do so (e.g. for the management of assets). This may be because of a lack of information about the figure of the *amministratore di sostegno* and his or her role. However, it may also be that many family members have reservations about starting a legal procedure that somehow limits the patient’s power, even if the goal is to protect the patient. In this sense, the suggestion that a legal representative be appointed just for the sake of participation in a research study, as well as being infeasible, seems hardly justified. Similar considerations can be extended to people who are competent at the time of enrolment in the research protocol but lose their capacity during the trial. Under the current Italian regulations, a legal representative should be appointed for those patients; however, there are likely to be difficulties with feasibility and family members’ willingness to start the legal procedure in this situation too.

### The case of uncertain capacity

The appointment of a legal representative does not answer the most interesting and difficult issue regarding the capacity of patients with AD; that is, the uncertain capacity of the subjects.

A diagnosis of dementia does not in itself mean that the subject is unable to understand and express valid informed consent. This view is often reflected in the literature, though in the routine provision of care it is often too easily assumed that people with dementia are incapable of making choices and decisions. Empirical studies that have assessed patients both in real-life conditions (i.e. during enrolment in clinical trials) and in hypothetical conditions using a range of instruments and methods for assessing research-related decision-making capacity [27] show that, although patients with AD perform worse than other patients and healthy subject control groups, they may be able to understand, appreciate, reason and express a valid choice when asked to take part in a research project [28-34]. Nevertheless, some patients have variable and borderline capacities and have been evaluated by researchers as ‘marginally competent’, ‘sufficiently competent’ [35] or ‘borderline competent’ [36]. Moreover, the judgement of competence/incompetence for patients with dementia is an especially difficult task and physicians may frequently disagree in their competency judgements, particularly in cases of mild to moderate dementia [37-39]. In addition, thresholds based on normative values or expert judgement can differ in terms of how many people are evaluated as incapable on at least one ability related to decision making [31], and agreement between standardised assessment methods and physicians’ judgement of competency may be poor for people with dementia [40]. However, research indicates that willingness to participate in research is similar for subjects with decisional impairment due to AD and healthy comparison subjects, showing that impaired subjects as a group are able to distinguish between research protocols with varying risk/benefit profiles [41]. Moreover, AD patients’ capacity performance may vary depending on the complexity and risk of trials, and even people evaluated as unable to give consent for research protocols may preserve the capacity to appoint a research proxy [32]. This suggests that competency is not a unitary concept or construct [42] and that while AD patients may lose the capacity to perform a specific act, they may retain the capacity for other affairs.

As the Nuffield Council on Bioethics [43] states in reference to individuals with dementia, ‘In many cases, it will be very clear whether a person with dementia does or does not have the capacity to make a particular decision. However, there will be times when the person’s ability to make a particular decision will be difficult to determine’. This ‘grey zone’ cannot be considered under the law, which assumes that a person either has or does not have the capacity to make a particular decision at a particular point in time; however, this is a very common situation, particularly in individuals in the earliest stages of dementia. Underestimating the uncertainty of subjects’ capacity may have significant consequences for patients who, depending on a physician’s judgement of competence, would be free to express their wishes and choose for themselves or, alternatively, would be subjected to resolutions taken for them by other people, which might result in their wishes not being fulfilled.

For subjects in the grey zone, in which capacity is uncertain and its judgement difficult, consent on the subject’s behalf may be the best solution to protect health professionals. However, we believe that this is not a good solution from an ethical perspective because it humiliates subjects and limits their potential to express themselves.

### The case of collaborative research

Opportunities to increase our prevention, diagnosis, treatment and care of people affected by AD can only be found in coordinated actions that unite the efforts of all those involved in this type of research. In Europe, the European Commission firmly invited countries ‘to pool their resources and better coordinate their research efforts in the field of neurodegenerative diseases, and Alzheimer’s in particular, by programming their research investments jointly for the first time, instead of each separately’ [44].

The fact that member states share the same regulations on clinical trials, which require (among other conditions) a legal representative in cases of incompetent adults, but have different legal procedures for the appointment of representatives, might delay research in some countries, such as Italy, where the procedure is burdensome. More importantly, this may be an obstacle to research on dementia and neurodegenerative disorders and may therefore inhibit the ability to make important discoveries.

### Suggestions for a possible solution

Informed consent should be regarded as a means to protect and promote the interests and rights of subjects within the medical sphere. A subject’s lack of capacity to give informed consent cannot solely be a sufficient reason to exclude that subject from participation in research. Such exclusion, far from protecting the subject, would prevent patients with AD from receiving possible benefits from research.

We have argued that the requirement for a legal representative may not be the best solution with respect to the research participation of patients with AD, even when it is quite clear that patients are unable to decide for themselves and, particularly, when their capacity is uncertain. Nevertheless, alternative measures that protect frail patients and that promote their interests and rights should be implemented.

#### The role of family members

Family members should play a major role in situations where it is impractical to appoint a legal representative merely for a subject’s inclusion in a research project and for subjects who are in the grey zone. This would also help to remove obstacles to collaborative research among European countries that do not share national legal requirements.

The possibility that a family member, even one not appointed by a judge as a legal representative, may make decisions on behalf of the patient needs careful consideration. From an ethical perspective, the most important requirement for a patient’s representative is that he or she has previously spent time and shared experiences with the patient and currently has a close relationship with them. This helps ensure that the representative is able to give a voice to the patient’s wishes and that the patient’s well-being is his or her primary concern. In this sense, the involvement of family members of patients with AD who have a poor understanding and decision-making capacity is valuable in ensuring that patients’ wishes and previous values and beliefs are respected [45]. For subjects with uncertain or variable capacity, ‘joint decision-making with trusted family members’ might help to bridge the gap between the point at which a person with dementia is fully able to make their own decisions and the time when formal proxy decision making becomes necessary on a regular basis [43]. This type of decision making may also bridge the gap between those periods when AD symptoms manifest more strongly and periods when they are weaker. The majority of clinical trials involving patients with AD require the active presence of a caregiver as an inclusion criterion. In most cases, the patients’ caregivers are members of the family. This means that family members are fully informed of the research protocol and are asked to monitor the patient during the trial and to collaborate with the research team in collecting information on the patient’s condition. We suggest that family members could be involved further and that researchers should trust that the opinions of family members regarding a patient’s participation in a clinical trial reflect what is most beneficial for an individual patient in a particular situation. This is also consistent with the law that established the *amministratore di sostegno* [46]. The law allows a hierarchy of family members to be appointed as legal proxy, including the beneficiary’s spouse, his or her partner, father or mother, son or daughter, brother or sister, or any other person who is close to the patient. Therefore, the law recognises that family members are the best legal representatives. Within the current legal framework, however, family members cannot sign the informed consent form on behalf of the patient unless they are the court-appointed legal representatives. Nevertheless, the information given to them and their opinions can be recorded. They can also sign a form confirming that they have received the information and have understood and appreciated it. This should be regarded, particularly by ethics committees, as proof of a prudent enrolment of patients in a clinical trial.

Our proposal to involve family members in the decision-making process is supported by empirical survey results. Family surrogate consent for AD research is supported both by the general public [47,48] and by people closer to the disease, such as caregivers and primary decision-makers for persons with dementia [49], and people at risk for AD [50]. A further argument in support of family members’ involvement in dementia research relates to the above-mentioned evidence that people suffering from AD can retain the capacity to appoint a proxy decision-maker even when they lose the capacity to give consent to research participation [32]. However, one study investigating consent practices of researchers in aging reported the soliciting of consent from family members to include people deemed incapable of consent in research protocols, even in jurisdictions where such authority is uncertain at law [51].

The involvement of family members can potentially extend the patient’s autonomy when it is compromised and does not exclude the patient from the decision-making process. In addition, patients would receive information according to their capacity for comprehension. This means that studies should provide straightforward, simple information and should avoid technical jargon and details that are not essential to a broad understanding of the research project. Furthermore, a patient’s refusal to participate in research would be respected. A risk nevertheless exists that proxies may persuade and influence patients, and may override patients’ desires in favour of what they consider is in the patient’s best interest [52]. Because of the specific Italian normative legal framework, we suggest that family members should also play a crucial role when the patient has previously expressed an advance directive.

#### The case of advance directives

Advance directives are an effective way to extend the autonomy of individuals who were previously able to decide for themselves but are currently incapable of decision making because of their pathological conditions. Advance directives are clearly limited because they cannot encompass all possible biomedical situations. Nevertheless, they may cover every aspect of research and treatment related to the subject’s health. These directives may include participation in biomedical research although this is uncommon [53,54] and efforts are needed to raise awareness about this matter [55]. Even now, advance directives in Italy have no legal value, although a discussion about them has begun in Parliament. Nonetheless, clear indications of the value and legitimacy of advance directives can be found in the Italian Deontological Code of the Order of Physicians [56]. These indications also feature in the Convention on Human Rights and Biomedicine [57] ratified into the Italian legislation and in the opinion of the Italian National Bioethics Committee on advance treatment statements [58], which suggests that the principle of advance directives has been accepted. We agree with the Italian Society of Neurology bioethics group [59] that advance directives are particularly valuable and effective for patients who have previously been deemed competent if they include the appointment of a trusted person who can contribute to the decision-making process in the context of current medical/scientific possibilities, on the basis of the indications given by the patient and his or her values and past life. The appointed trusted person should be involved in decisions about research participation even when explicit choices on the matter are not included in the patient’s directives, as her appointment shows unambiguously the patient’s trust and will to be represented. In the context of advance directives, the patient’s family member, whether explicitly appointed as trusted person or not appointed, would play a crucial role in respecting those directives.

#### The role of the research ethics committees

The involvement of family members in the decision-making process is not a substitute for other requirements for the implementation of biomedical research involving patients with poor or uncertain capacity. These requirements set out the conditions for offers of research participation to such patients and should be fulfilled. They specify the following: that the proposed research is essential to validate data; such research relates directly to the subject’s clinical condition; clinical trials have been designed to minimise pain, discomfort, fear and any other foreseeable risk; and the expected benefit will outweigh the risks or the trial will produce no risk at all [11]. The requirements for good communication with the patient and for the clinical assessment of capacity should also be fulfilled. All of these requirements should be considered first by the research sponsor during the planning of the research protocol and then by the ethics committee, whose role is to ‘protect the dignity, rights, safety and well-being of research participants’ [12].

With regard to the family members of patients who are invited to take part in a research study, ethics committees should acknowledge their role as persons who extend the autonomy of patients and who are strongly interested in the patient’s health and well-being. In this sense, ethics committees should accept family members’ opinions about the patient’s participation in a clinical trial as valid decisions regarding the patient’s enrolment.

To ensure that every effort to respect patients’ autonomy and wishes has been made [60], ethics committees should require research protocols to describe the planned informed consent process, as follows: the methods and instruments used to assess patients’ competence, the possible presence of an independent evaluator of competence and an independent auditor of the informed consent process, the method used to identify the patients’ representatives, and the value given to advance directives expressed by patients when they were fully competent. We recognise that providing an independent evaluator of competence and an auditor of informed consent might be difficult, although not impossible, at least in qualified centres. Ethics committees should nevertheless be informed about the planned consent process, and they would evaluate on a case-by-case basis if this provides sufficient protection for the prospective participants. Last, members of research ethics committees might also personally supervise the enrolment of patients in situations where they judge this is important, even though supervision requires human resources that ethics committees rarely have.

It is widely assumed that not every kind of research deserves the same level of subject protection. This should be judged according to the benefit/risk ratio of the research protocols; that is, the level of protection should be progressively higher in relation to an increase in risks and a decrease in expected benefits. On the basis of ethical recommendations [61] and the ethics committees’ evaluation, some of the above suggestions could be deemed essential while others could be deemed excessive and discounted. However, in our opinion, the requirement to involve patients’ family members in the biomedical research consent process should be preserved.

## Summary

In accordance with the National Bioethics Advisory Commission report [61], we stress the importance of research aimed to increase knowledge of the decision-making capacity of individuals with mental disorders, the best ways of assessing that capacity, and the methods that can improve the process of informed consent. At the same time, we consider that concerns about the difficulty of obtaining fully valid consent of patients with AD should not prevent them from potentially taking part in clinical trials and benefiting from scientific progress. We argue that the requirement for patients to have a legal representative may not be the best solution in all countries and clinical situations and suggest that the role of patients’ family members in the decision-making process should be promoted. In addition, we highlight the possible role of advance directives and ethics committees.
